# First person – Victoria Hyland

**DOI:** 10.1242/bio.061927

**Published:** 2025-02-25

## Abstract

First Person is a series of interviews with the first authors of a selection of papers published in Biology Open, helping researchers promote themselves alongside their papers. Victoria Hyland is first author on ‘ Ccn2a acts downstream of *cx43* to influence joint formation during zebrafish fin regeneration’, published in BIO. Victoria is a PhD Student in the lab of Dr M. Kathryn Iovine at Bethlehem, Pennsylvania, USA, investigating the tight coordination of skeletal patterning in zebrafish.


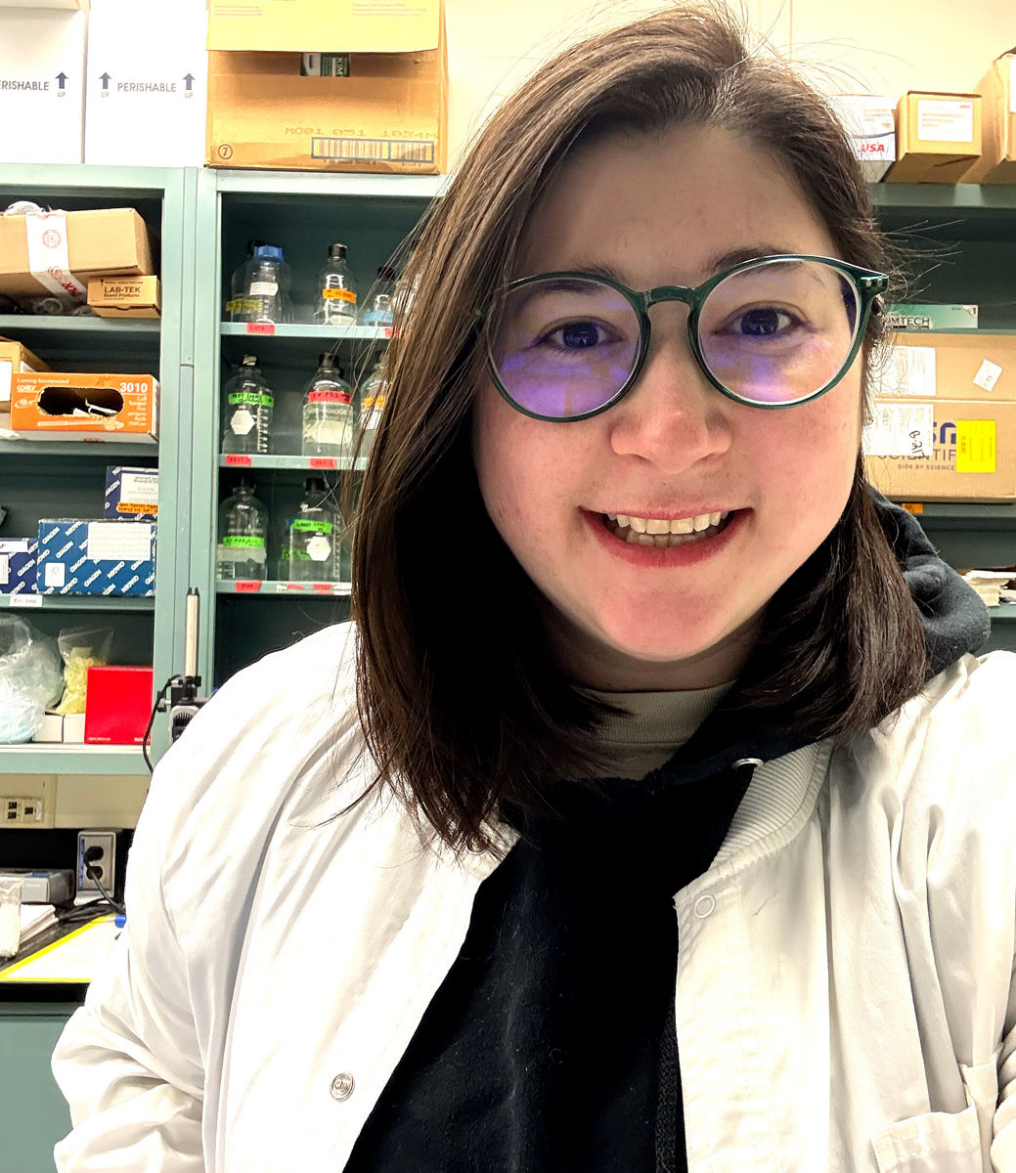


**Victoria Hyland**

**Describe your scientific journey and your current research focus**

My journey has been shaped by a strong interest in understanding biological systems, a commitment to research, and a desire to uncover the molecular mechanisms behind complex processes. I first began my academic path as an undergraduate at the University of Scranton, where I first gained hand-on experience in a research lab. I then pursued a Master's degree at Lehigh University in molecular biology, which helped to deepen my understanding of biological systems. After my Master's degree, I went on to work at Penn State Cancer Institute working on clinical trials with lung cancer patients. I was able to gain firsthand insight into how research translates into patient care. I found the work rewarding, but it also sparked a deeper curiosity on the fundamental biological mechanisms that weren't fully understood. This realization pushed me toward my PhD back at Lehigh University. Now I am studying skeletal patterning during regeneration in zebrafish focusing on how specific molecular signals regulate bone and joint formation.

**Who or what inspired you to become a scientist?**

I've always been curious about how things work, but what really inspired me to pursue science was the idea of making discoveries that could help people. I initially sought out to be a medical doctor as an undergrad, but doing research in Dr Gomez's lab at the University of Scranton changed my view on science and solidified my desire to become a scientist.

I've always been curious about how things work

**How would you explain the main finding of your paper?**

Regrowing body parts, like a lizards tail or a zebrafish fins, requires precise control over when and where bones and joints form. The protein Cx43 helps control the timing of joint formation by preventing joints from forming too early. This paper found that another protein, Ccn2a, also plays a role in this process by working after Cx43 to further regulate joint development. It was further found that signals from Beta-Catenin and Yap also influence Ccn2a, adding more layers of control to how joints are formed. By uncovering these details, this paper provides new insights into how bones and joints regenerate, which could have future applications in treating joint diseases.

this paper provides new insights into how bones and joints regenerate

**Figure:**
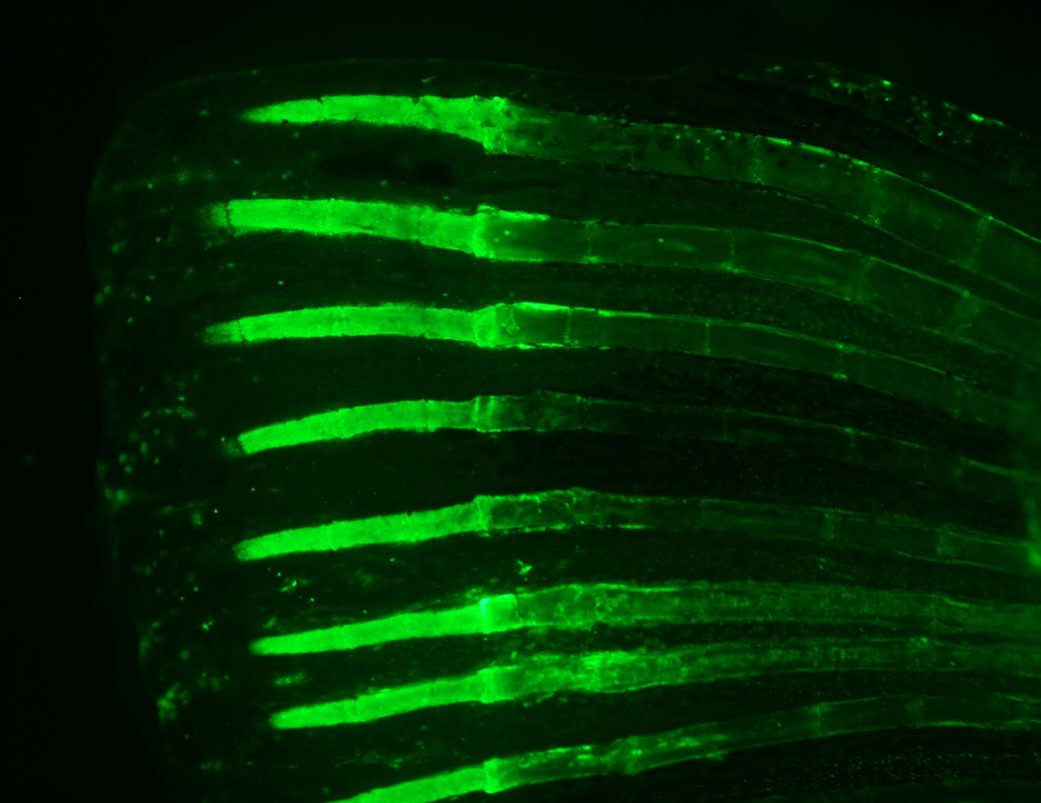
**A calcein-stained zebrafish fin during regeneration.** This enables you to visualize the bones and joints.

**What are the potential implications of this finding for your field of research?**

Understanding how proteins like Cx43 and CCn2a regulate joint formation in zebrafish could help scientists develop new treatments for conditions like osteoarthritis or joint injuries, where cartilage and bone regeneration are limited. If similar pathways exist in human researchers may be able to prevent joint degeneration using targeted therapies.

**What do you enjoy most about being an early-career researcher?**

As an early career researcher, what I enjoy the most is the excitement of discovering new things and knowing that my work is contributing to something bigger. The problem-solving aspect is always challenging, but it's satisfying when things finally click and I see the results of my work come together.

**What's next for you?**

What's next for me is finishing my PhD at Lehigh University and completing my research on joint formation in zebrafish. As I approach graduation, I'm focused on transitioning into a career in medical writing.
